# Response surface modeling for hot, humid air decontamination of materials contaminated with *Bacillus anthracis* ∆Sterne and *Bacillus thuringiensis* Al Hakam spores

**DOI:** 10.1186/s13568-014-0021-3

**Published:** 2014-05-01

**Authors:** Edward J Prokop, John R Crigler, Claire M Wells, Alice A Young, Tony L Buhr

**Affiliations:** 1 Naval Surface Warfare Center, Dahlgren Division, CBR Concepts and Experimentation Branch, 4045 Higley Road Suite 345, Dahlgren VA 22448-5162, USA

**Keywords:** Bacillus, Spore, Decontamination, Hot humid air, RSM

## Abstract

Response surface methodology using a face-centered cube design was used to describe and predict spore inactivation of *Bacillus anthracis* ∆Sterne and *Bacillus thuringiensis* Al Hakam spores after exposure of six spore-contaminated materials to hot, humid air. For each strain/material pair, an attempt was made to fit a first or second order model. All three independent predictor variables (temperature, relative humidity, and time) were significant in the models except that time was not significant for *B. thuringiensis* Al Hakam on nylon. Modeling was unsuccessful for wiring insulation and wet spores because there was complete spore inactivation in the majority of the experimental space. In cases where a predictive equation could be fit, response surface plots with time set to four days were generated. The survival of highly purified *Bacillus* spores can be predicted for most materials tested when given the settings for temperature, relative humidity, and time. These predictions were cross-checked with spore inactivation measurements.

## Introduction

Conventional chemical decontamination (e.g. bleach and/or harsh oxidizers) can create health risks and damage many materials, which increases decontamination training costs, personal protection requirements, and may result in replacement, reconstruction and/or remodeling of sensitive equipment and materials. These issues increase decontamination costs and the cleanup time required after decontamination (Craig and Anderson [1995]; Garverick [1994]; Rutala et al. [2008]; Koch et al. [2001]; Herzberg [2012]). For example, decontamination and site remediation following the 2001 anthrax (*B. anthracis*) attacks cost in excess of 300 million US dollars and took 34 months to complete (Canter [2005]; Buhr et al. [2012a]). Aircraft are of particular concern since they are a vehicle for global epidemic spread and are comprised of extremely sensitive equipment and materials (Moser et al. [1979]; Olsen et al. [2003]). Because there are limited choices for aircraft interior decontamination, corrosive chemicals may be improperly selected and used in the event of potential international epidemics such as influenza or SARS. The use of corrosive chemicals may then undermine the integrity of the aircraft. Thus, there is a need to develop effective decontaminants with low corrosiveness and improved materials compatibility for various materials including, but not exclusive to those found on aircraft.

Thermal inactivation of biological microbes is a well-known physical procedure that applies to both food sterilization and decontamination of inanimate objects. The Roman poet Virgil described attempts at thermal decontamination of *B. anthracis*-infected wool and hides several millennia ago (Dirckx [1981]; Sternbach [2003]). Cooking, grilling and pasteurization are used to inactivate food microbes (Ullmann [2007]). Autoclaving is another well-known thermal inactivation procedure (Hugo [1991]). Numerous manuscripts have been published describing thermal inactivation of *Bacillus* spores (Murrell and Scott [1966]; Alderton and Snell [1970]; Gerhardt and Marquis [1989]; Melly et al. [2002]; Coleman et al. [2007]; Sunde et al. [2009]; Zhang et al. [2010]; Buhr et al. [2012b]).

Three variables to control thermal decontamination are time, temperature and water content. Adjustment of these variables may be less damaging to materials compared to application of corrosive chemicals (Decker et al. [1954]; Ehrlich et al. [1970]; Holwitt et al. [2000]). Response surface methodology (RSM) (Beauregard et al. [1992]; Myers et al. [2009]) was coupled with the development of test methods to control time, temperature and relative humidity while safely containing spores (Buhr et al. [2012b]). Analogous to slow cooking, spore inactivation of spore-contaminated materials was tested using low temperatures and long times in order to explore the limits of sporicidal efficacy using hot, humid air. Six materials were chosen that were representative of materials found on aircraft: wiring insulation, polypropylene, aircraft performance coating on aluminum, nylon, InsulFab insulation and anti-skid tape on aluminum (Buhr et al. [2012b]). Decontamination of these materials using hot, humid air showed comparable spore kill kinetics among virulent *B. anthracis* Ames, attenuated *B. anthracis* ∆Sterne and *B. thuringiensis* Al Hakam spores. In order to mitigate decontamination test costs and schedule associated with BSL3 testing of *B. anthracis* Ames in a large-scale design of experiments, *B. anthracis* ∆Sterne and *B. thuringiensis* Al Hakam spores were selected for decontamination testing that measured decontamination on greater than 2,500 tests and controls with ≥7 logs of spores per test (Buhr et al. [2012b]). This manuscript focuses on describing the application of response surface methodology to experimental design and data analysis of the hot, humid air data. The objective is to mathematically describe the effectiveness of hot, humid air decontamination using first or second order polynomial surfaces and to determine the region of inactivating 6 logs of spores using the lower bound of a 90% confidence interval for the surface. This threshold was selected because it is a requirement for the Food and Drug Administration, Environmental Protection Agency and Department of Defense (Tomasino et al. [2008]; Ryan et al. [2010]; Buhr et al. [2012a]). The result of this research is a mathematical description that can help guide an end-user to find acceptable operating conditions depending on the material(s) to be decontaminated.

## Materials and methods

### *Bacillus anthracis* ∆Sterne and *Bacillus thuringiensis* Al Hakam spores

*Bacillus anthracis* ∆Sterne was obtained from the Unified Culture Collection at USAMRIID (Frederick, MD, USA): Unified Culture Collection Identifier BAC1056, lot number CA062700A. *Bacillus thuringiensis* Al Hakam was provided by Johnathan Kiel at Brooks Air Force Base, San Antonio, TX (Buhr et al. [2008-b]; McCartt et al. [2011]).

### Coupon materials and inoculation

All substrates and substrate inoculations have been previously described (Buhr et al. [2012b]) and are summarized here. A substrate was defined as an individual coupon, polypropylene or wet sample. The aircraft-related materials chosen for this study were aluminum 2024-T3 coated with Aircraft Performance Coating (APC), aluminum 2024-T3 coated with anti-skid material, InsulFab insulation, wiring insulation, nylon webbing, and polypropylene. Each substrate was cut into 2×2 centimeter squares except polypropylene. All substrates were inoculated with ≥7 logs of spores per substrate. Spores were dried inside 50 ml conical tubes for polypropylene tests. An additional seventh substrate was wet spores; i.e., spores in 0.1% Tween 80, but never dried onto a surface.

### Environmental test chamber setup and RSM

The environmental chamber validation and RSM have been previously described (Buhr et al. [2012b]) and are summarized here. Envirotronics (Grand Rapids, MI) LH0010 environmental test chambers were used to control temperature and relative humidity during testing. Three test conditions were selected for each of the three test parameters of temperature, relative humidity and time. The highest (strongest) selected combination of conditions was expected to result in complete spore inactivation, the lowest (mildest) selected combination of conditions was expected to result in complete spore survival, and the midpoint selected was expected to result in partial spore inactivation. The test conditions were equally spaced in an attempt to generate a spore inactivation response curve for each strain/substrate combination. The temperature limits considered took into account the goal of completely inactivating spores at the lowest temperature possible in order to meet materials temperature qualification limits. Based on preliminary decontamination test screening of virulent *B. anthracis* Ames, attenuated *B. anthracis* ∆Sterne and *B. thuringiensis* Al Hakam spores, the upper limit for temperature and relative humidity was set at 77°C, 90% relative humidity because the data indicated that complete spore inactivation would be achieved within 7 days at this combination. For the lower limits, the temperature was set at 60°C, 60% relative humidity, conditions where spores were known to survive for multiple days. Initially, temperatures of 140, 155 and 170 °F were selected, then converted to Celsius and rounded to 60, 68 and 77°C. The final test conditions for relative humidity were 60, 75, and 90%. The final conditions for time were 1, 4, and 7 days. Each temperature/relative humidity combination was above the dew point to prevent condensation.

### Test methods and control-driven experimental design

Test methods and experimental design have been previously described (Buhr et al. [2012b]) and are summarized here. Test methods were developed to contain spores or spore-inoculated substrates within filter-capped 50 ml conical tubes. The 0.2 *μ*M filters permitted hot, humid air to pass through the filter cap and the filter containing spores. This method was critical for proof-of-principle testing with virulent *B. anthracis* Ames spores since a fan circulated hot, humid air during testing. This method also permitted spore extraction in the test tube without extra handling steps of the spore-inoculated substrate.

For each test run, spores (≥7 logs of spores) from each of ten independent spore preparations (five spore preparations for *B. anthracis* ∆Sterne spores and another five spore preparations for *B. thuringiensis* Al Hakam) were inoculated on the test substrates and the control substrates. Spore survival was quantified for 70 tests and 70 room temperature controls for each test run with a total of 19 independent test runs. For each test run, there were ten substrates for each of the six materials plus the wet control (7 total) for test samples, and the six materials plus the wet control (7 total) for the room temperature control samples. There were a total of 1,330 independent tests and 1,330 independent room temperature controls for the entire study.

### Quantification of spore survival and calculations

Spore quantification and spore survival calculations have been described (Buhr et al. [2012b]), and are summarized here. Spore-inoculated substrates were exposed to specified temperature, relative humidity and time, or left at 22 ± 3°C and ambient relative humidity. After decontamination, spores were extracted in ten ml of extraction medium followed by dilution plating and scoring for growth. Wet spore controls, incubated at 22 ± 3°C and ambient relative humidity, served as the 100% spore recovery reference values in order to correct for spore extraction efficiency from different substrates. The number of surviving spores (CFU ml−1) from each hot, humid air-treated test substrate was divided by the extraction percentage for that substrate in order to determine the number of surviving spores (CFU ml−1). This spore concentration was multiplied by 10 ml to determine the total number of spores surviving (total CFU) for each test sample. A log_10_ transformation (log_10_ (total CFU + 1)) of the total surviving spores was then calculated.

### Response surface methodology and test design

Response surface methodology is a statistical procedure used to understand, improve, and optimize a process. The objective is to model a response variable in terms of one or more independent predictor variables. Second order polynomial models (Equation 1)

(1) $$y=\beta_{0}+\sum_{j=1}^{k}\beta_{j}x_{j}+\sum_{j=1}^{k}\beta_{\mathrm{jj}}x_{j}^{2}+\sum_{i<j}\sum_{j=2}^{k}\beta_{\mathrm{ij}}x_{i}x_{j}$$

are most commonly used in RSM because they are flexible in describing data in which there is curvature. They also make for straightforward locating of optima. RSM is typically used sequentially. An experiment is first done to screen potential predictor variables. If it is determined that the current settings of the predictors are near an optimum, supplementary runs are done to fit a second order model. If the experimental region is not near an optimum, the method of steepest ascent can be used to find the optimum or arrive at a region of operability should an optimum be unattainable. In other situations, the region of operability is the entire experimental region. Such was the case for this study. The experiment used in this situation was the face-centered cube design (FCD) (Myers and Montgomery [2002]). The final design in terms of coded and original predictor variables is given in Table 1.

**Table 1:** Face-centered cube design matrix with coded and original units

**Run**	**X**_**1**_	**X**_**2**_	**X**_**3**_	**Temperature (°C)**	**Rel. humidity (%)**	**Days**	**Block**
3	−1	−1	−1	60.0	60	1	0
9	−1	−1	1	60.0	60	7	0
12	−1	1	−1	60.0	90	1	0
6	−1	1	1	60.0	90	7	0
1	0	0	0	68.3	75	4	0
4	0	0	0	68.3	75	4	0
8	0	0	0	68.3	75	4	0
11	0	0	0	68.3	75	4	0
13	0	0	0	68.3	75	4	0
2	1	−1	−1	76.7	60	1	0
10	1	−1	1	76.7	60	7	0
7	1	1	−1	76.7	90	1	0
5	1	1	1	76.7	90	7	0
14	−1	0	0	60.0	75	4	1
16	0	−1	0	68.3	60	4	1
18	0	0	−1	68.3	75	1	1
19	0	0	1	68.3	75	7	1
17	0	1	0	68.3	90	4	1
15	1	0	0	76.7	75	4	1

As Table 1 shows, there were three predictors, temperature (T), relative humidity (RH), and days (D). These were transformed to the coded variables, *x*_1_, *x*_2_, and *x*_3_ in order to better determine the relative size of their effects. The codes were $x_{1}=\frac{T-68.\bar{3}}{8.\bar{3}}x_{2}=\frac{\mathrm{RH}-75}{15}\;\mathrm{and}\;x_{3}=\frac{D-4}{3}.$

The measured response, p(x1, x2, x3), was the proportion of spores inactivated divided by the average number of spores tested. Since the response was a proportion, the proper link function is the logit (or log odds) (Agresti [1996,2002]; Hosmer and Lemeshow [2000]); i.e., $y=\log\left(\frac{p}{1-p}\right)$ where p is shorthand for p(x1, x2, x3) and log denotes the natural logarithm function. If a particular material had complete spore inactivation on one or two observations, p was conservatively imputed as the minimum detection level, $\left(\frac{0.1}{\;\mathrm{avg}.\;\mathrm{no}.\;\mathrm{spores}\;\mathrm{tested}\;}\right)$, to bound the logit away from infinity. In some cases, however, too many coupons had complete inactivation and could not be modeled using Equation 1.

An attempt to fit a second order model was made for each strain on each material. ANOVA tables for the sequential sum of squares were used to determine which group of terms contributed significantly to the models. The groups were first-order terms (*x*_1_, *x*_2_, *x*_3_), pure quadratic terms (*x*^2^_1_, *x*^2^_2_, *x*^2^_3_), and interaction terms (*x*_1_*x*_2_, *x*_1_*x*_3_, *x*_2_*x*_3_). If a group of terms was deemed significant, all terms from that group were kept in the model (Lenth [2009]). A blocking variable, *b*, was included in each model since the observations were gathered in two phases, one phase for the factorial and center points, the second phase for the face-centered points. Information concerning individual parameter estimates, standard error and statistical significance is provided in separate ANOVA tables.

Each model was subjected to diagnostic measures consisting of the F-test for lack of fit, examination of raw residuals and jackknifed residuals vs. fitted values, and examination of normal plots based on both raw residuals and jackknifed residuals (Venables and Ripley [1999]). These diagnostics were crucial for outlier detection and in determining for which cases a model could not be fit. Analysis was performed using R along with the rsm package (Lenth [2009]).

## Results

In the sections that follow equations were fitted for each strain/substrate combination and graphically compare the two strains when possible. There is no physical reason to use four days when contrasting the graphs other than that it is the median value for days. Likewise, there is no particular reason to fix days and let temperature and relative humidity vary other than to keep the manuscript concise. The interested reader can use their favorite software and the fitted equations to construct whatever graphs he or she wishes.

### Aircraft Performance Coating on aluminum (APC)

#### *B. anthracis* ∆Sterne on APC

**Figure 1:**
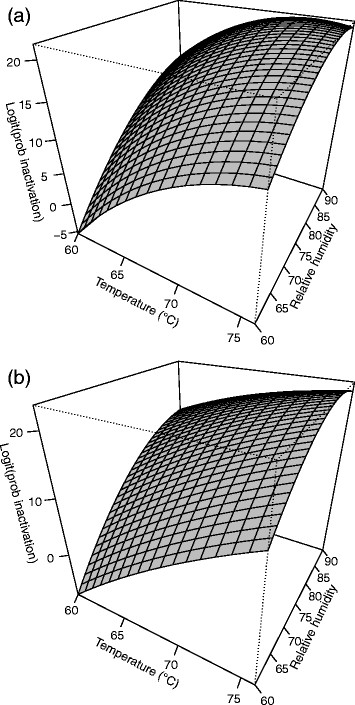
**Response surface plot for (a)** ***B. anthracis*** **∆Sterne and (b)** ***B. thuringiensis*** **Al Hakam on APC at 4 days.**

All test variables were at the lowest settings during test run three, and there was no or minimal spore inactivation. This observation could not be modeled using Equation 1. Hence, data from test run three was omitted and the model was refitted. A second order model with pure quadratic terms and two-way interactions was needed. The F-test for lack of fit had a P-value of 0.110 which was large enough to not reject the model. R^2^ and adjusted R^2^ for the model were 0.9548 and 0.8901, respectively. Table 2 shows the parameter estimates, standard errors, t-values, and P-values. All second order terms were significant. The fitted equation for inactivation of *B. anthracis* ∆Sterne on APC is *Ŷ* = 17.5984 − 0.7847*b* + 6.7224*x*_1_ + 6.2954*x*_2_ + 6.7968*x*_3_ − 2.6850*x*_1_*x*_2_ − 3.4490*x*_1_*x*_3_ − 2.8576*x*_2_*x*_3_ − 3.6926*x*^2^_1_ − 3.3049*x*^2^_2_ − 2.7348*x*^2^_3_, where *Ŷ* is the log odds of spore inactivation. Figure 1 shows a slice of the response surface at four days and block (b) fixed at its mean value of 0.3333.

**Table 2:** **Parameter estimates for** ***B. anthracis*** **∆Sterne and** ***B. thuringiensis*** **Al Hakam on APC**

**Model term**	**Parameter estimate**	**Standard error**	**t-value**	**P-value**
*B. anthracis* ∆Sterne				
Intercept	17.5984	0.9562	18.405	3.4646 × 10^−7^
Block (b)	−0.7847	1.1276	−0.696	0.5089
*x*_1_	6.7224	0.8281	8.118	8.3006 × 10^−5^
*x*_2_	6.2954	0.8281	7.602	0.0001
*x*_3_	6.7968	0.8281	8.208	7.7356 × 10^−5^
*x*_1_*x*_2_	−2.685	0.9636	−2.786	0.0271
*x*_1_*x*_3_	−3.449	0.9636	−3.579	0.0090
*x*_2_*x*_3_	−2.8576	0.9636	−2.965	0.0209
*x*^2^_1_	−3.6926	1.3148	−2.809	0.0262
*x*^2^_2_	−3.3049	1.3148	−2.514	0.0402
*x*^2^_3_	−2.7348	1.3148	−2.08	0.0761
*B. thuringiensis* Al Hakam				
Intercept	16.8775	1.5466	10.912	1.1998 × 10^−5^
Block (b)	−0.7869	1.8239	−0.431	0.6791
*x*_1_	7.5240	1.3394	5.617	0.0008
*x*_2_	7.5517	1.3394	5.638	0.0008
*x*_3_	7.2767	1.3394	5.433	0.0010
*x*_1_*x*_2_	−3.6367	1.5587	−2.333	0.0524
*x*_1_*x*_3_	−3.5525	1.5587	−2.279	0.0567
*x*_2_*x*_3_	−2.7313	1.5587	−1.752	0.1232
*x*^2^_1_	−1.4004	2.1266	−0.658	0.5313
*x*^2^_2_	−4.6715	2.1266	−2.197	0.0640
*x*^2^_3_	−3.3409	2.1266	−1.571	0.1602

Df = 1 for estimates.

#### *B. thuringiensis* Al Hakam on APC

All test variables were at the lowest settings during test run three, and there was no or minimal spore inactivation. This observation could not be modeled using Equation 1. Hence, data from test run three was omitted and the model was refitted. A second order model with pure quadratic terms and two-way interactions was needed. The F-test for lack of fit had a P-value of 0.289 which does not indicate lack of fit. R^2^ and adjusted R^2^ for the model were 0.9079 and 0.7763, respectively. Table 2 shows the parameter estimates, standard errors, t-values, and P-values. The fitted equation for inactivation of *B. thuringiensis* Al Hakam on APC is *Ŷ* = 16.8775 − 0.7869*b* + 7.5240*x*_1_ + 7.5517*x*_2_ + 7.2767*x*_3_ − 3.6367*x*_1_*x*_2_ − 3.5525*x*_1_*x*_3_ − 2.7313*x*_2_*x*_3_ − 1.4004*x*^2^_1_ − 4.6715*x*^2^_2_ − 3.3409*x*^2^_3_, where *Ŷ* is the log odds of spore inactivation. Figure 1 shows a slice of the response surface at four days and block (b) fixed at its mean value of 0.3333.

### Antiskid

#### *B. anthracis* ∆Sterne on antiskid

**Figure 2:**
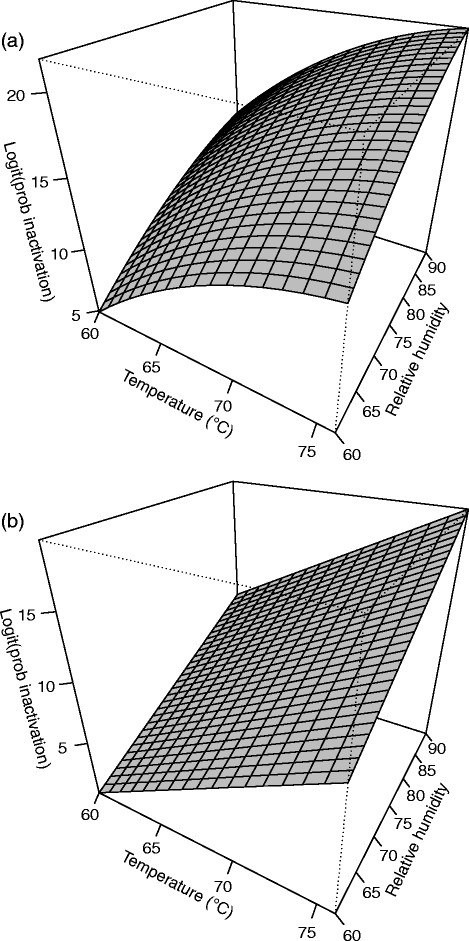
**Response surface plot for (a)** ***B. anthracis*** **∆Sterne and (b)** ***B. thuringiensis*** **Al Hakam on antiskid at 4 days.**

A second order model with pure quadratic terms was needed. Two-way interaction terms did not contribute significantly to the model (p = 0.846) and were not included. The F-test for lack of fit had a P-value of 0.270 which did not indicate lack of fit. R^2^ and adjusted R^2^ for the model were 0.8833 and 0.8091, respectively. Table 3 shows the parameter estimates, standard errors, t-values, and P-values. The pure quadratic term for days is the significant second order term. The fitted equation for inactivation of *B. anthracis* ∆Sterne on antiskid is *Ŷ* = 16.4509 − 0.9795*b* + 4.4938*x*_1_ + 3.9625*x*_2_ + 3.8248*x*_3_ − 1.7352*x*^2^_1_ − 1.0571*x*^2^_2_ − 2.8849*x*^2^_3_, where *Ŷ* is the log odds of spore inactivation. Figure 2 shows a slice of the response surface at four days and block (b) fixed at its mean value of 0.3158.

**Table 3:** **Parameter estimates for** ***B. anthracis*** **∆Sterne and** ***B. thuringiensis*** **Al Hakam on antiskid**

**Model term**	**Parameter estimate**	**Standard error**	**t-value**	**P-value**
*B. anthracis* ∆Sterne				
Intercept	16.4509	1.2098	13.598	3.1872 × 10^−8^
Block (b)	−0.9795	1.4042	−0.698	0.5000
*x*_1_	4.4938	0.8555	5.253	0.0003
*x*_2_	3.9625	0.8555	4.632	0.0007
*x*_3_	3.8248	0.8555	4.471	0.0009
*x*^2^_1_	−1.7352	1.6443	−1.055	0.3139
*x*^2^_2_	−1.0571	1.6443	−0.643	0.5335
*x*^2^_3_	−2.8849	1.6443	−1.755	0.1071
*B. thuringiensis* Al Hakam				
Intercept	8.9575	0.6919	12.946	8.3652 × 10^−9^
Block (b)	3.2641	1.1984	2.724	0.0174
*x*_1_	5.2788	0.7579	6.965	9.8472 × 10^−6^
*x*_2_	4.4240	0.7579	5.837	5.8096 × 10^−5^
*x*_3_	3.7842	0.7579	4.993	0.0002

Df = 1 for estimates.

#### *B. thuringiensis* Al Hakam on antiskid

Run number one, a center point, was difficult to model and an outlier in the residual and normal plots. This run was omitted and the model refitted. Only a first order model was needed. Neither two-way interactions nor pure quadratic effects were significant (p = 0.560 and p = 0.513, respectively). The F-test for lack of fit had a P-value of 0.652 which did not indicate lack of fit. R^2^ and adjusted R^2^ for the model were 0.8984 and 0.8671, respectively. Table 3 shows the parameter estimates, standard errors, t-values, and P-values. The fitted equation for inactivation of *B. thuringiensis* Al Hakam on antiskid is *Ŷ* = 8.9575 + 3.2641*b* + 5.2788*x*_1_ + 4.4240*x*_2_ + 3.7842*x*_3_, where *Ŷ* is the log odds of spore inactivation. Figure 2 shows a slice of the response surface at four days and block (b) fixed at its mean value of 0.3333.

### InsulFab

#### *B. anthracis* ∆Sterne on InsulFab

**Figure 3:**
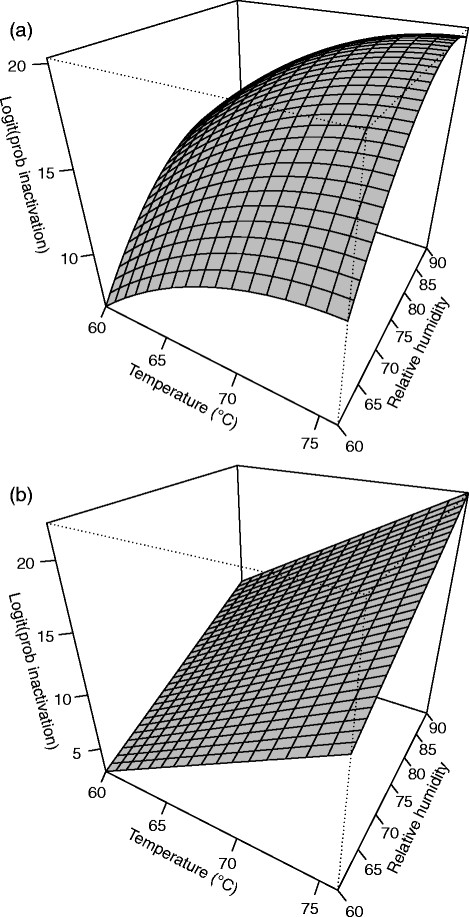
**Response surface plot for (a)** ***B. anthracis*** **∆Sterne and (b)** ***B. thuringiensis*** **Al Hakam on InsulFab at 4 days.**

Run number one, a center run, was an outlier and deleted from the analysis. A second order model with pure quadratic terms was used in this case. Two-way interaction terms did not contribute significantly to the model (p = 0.890) and were not included. The F-test for lack of fit had a P-value of 0.261 which did not indicate lack of fit. R^2^ and adjusted R^2^ for the model were 0.8275 and 0.7068, respectively. Table 4 shows the parameter estimates, standard errors, t-values, and P-values. The pure quadratic term for relative humidity is the significant second order term. Although 0.176 is a somewhat high P-value for significance, it is preferable to allow for the possibility of some curvature of the response surface. The fitted equation for inactivation of *B. anthracis* ∆Sterne on InsulFab is *Ŷ* = 17.0315 − 2.0862*b* + 3.0930*x*_1_ + 3.5849*x*_2_ + 3.7208*x*_3_ − 1.6774*x*^2^_1_ − 2.9997*x*^2^_2_ − 1.6517*x*^2^_3_, where *Ŷ* is the log odds of spore inactivation. Figure 3 shows a slice of the response surface at four days and block (b) fixed at its mean value of 0.3333.

**Table 4:** **Parameter estimates for** ***B. anthracis*** **∆Sterne and** ***B. thuringiensis*** **Al Hakam on InsulFab**

**Model term**	**Parameter estimate**	**Standard error**	**t-value**	**P-value**
*B. anthracis* ∆Sterne				
Intercept	17.0315	1.6808	10.1330	1.4082 × 10^−6^
Block (b)	2.0862	1.8155	1.1490	0.2772
*x*_1_	3.0930	1.0630	2.9010	0.0156
*x*_2_	3.5849	1.0630	3.3720	0.0071
*x*_3_	3.7208	1.0630	3.5000	0.0057
*x*^2^_1_	−1.6774	2.0586	−0.815	0.4341
*x*^2^_2_	−2.9997	2.0586	−1.457	0.1757
*x*^2^_3_	−1.6517	2.0586	−0.802	0.4410
*B. thuringiensis* Al Hakam				
Intercept	12.0283	1.0831	11.1050	2.5117 × 10^−8^
Block (b)	2.1611	1.9274	1.1210	0.2810
*x*_1_	5.7042	1.2349	4.6190	0.0004
*x*_2_	4.3161	1.2349	3.4950	0.0036
*x*_3_	3.7813	1.2349	3.0620	0.0084

Df = 1 for estimates.

#### *B. thuringiensis* Al Hakam on InsulFab

Only a first order model was needed. Neither two-way interactions nor pure quadratic effects were significant (p = 0.9997 and p = 0.9386, respectively). The F-test for lack of fit had a P-value of 0.340 which did not indicate lack of fit. R^2^ and adjusted R^2^ for the model were 0.7594 and 0.6906, respectively. Table 4 shows the parameter estimates, standard errors, t-values, and P-values. The fitted equation for inactivation of *B. thuringiensis* Al Hakam on Insulfab is *Ŷ* = 12.0283 + 2.1611*b* + 5.7042*x*_1_ + 4.3161*x*_2_ + 3.7813*x*_3_, where *Ŷ* is the log odds of spore inactivation. Figure 3 shows a slice of the response surface at four days and block (b) fixed at its mean value of 0.3158.

### Nylon

#### *B. anthracis* ∆Sterne on nylon

**Figure 4:**
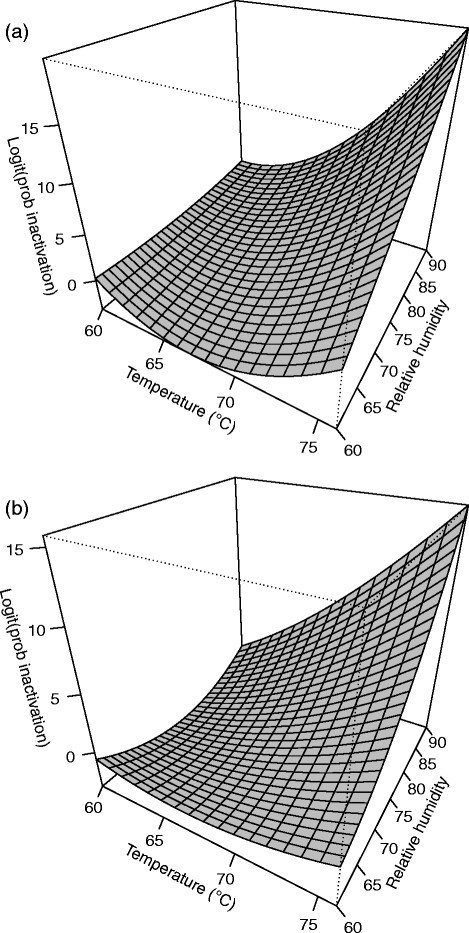
**Response surface plot for (a)** ***B. anthracis*** **∆Sterne and (b)** ***B. thuringiensis*** **Al Hakam on nylon at 4 days.**

*B. anthracis* ∆Sterne spores on nylon were the most difficult to inactivate and to model. Using residual plots, three problematic test runs, one, three and fifteen, were identified. These observations were omitted and the model refitted with the caveat that the results for nylon are not as dependable as the results for other coupons. A second order model with pure quadratic terms and two-way interactions was needed. The F-test for lack of fit had a P-value of 0.141 which did not indicate lack of fit. R^2^ and adjusted R^2^ for the model were 0.9972 and 0.9917, respectively. Table 5 shows the parameter estimates, standard errors, t-values, and P-values. The fitted equation for inactivation of *B. anthracis* ∆Sterne on nylon is *Ŷ* = 0.07582 + 2.1487*b* + 6.0157*x*_1_ + 4.6424*x*_2_ + 1.3926*x*_3_ + 4.2282*x*_1_*x*_2_ − 0.8748*x*_1_*x*_3_ − 0.3612*x*_2_*x*_3_ + 5.3117*x*^2^_1_ + 0.6467*x*^2^_2_ − 2.3881*x*^2^_3_, where *Ŷ* is the log odds of spore inactivation. Figure 4 shows a slice of the response surface at four days and block (b) fixed at its mean value of 0.3125.

**Table 5:** **Parameter estimates for** ***B. anthracis*** **∆Sterne and** ***B. thuringiensis*** **Al Hakam on nylon**

**Model term**	**Parameter estimate**	**Standard error**	**t-value**	**P-value**
*B. anthracis* ∆Sterne				
Intercept	0.0758	0.3034	0.2500	0.8126
Block (b)	2.1487	0.3841	5.5930	0.0025
*x*_1_	6.0157	0.2809	21.4170	4.1152 × 10^−6^
*x*_2_	4.6424	0.2405	19.3010	6.8867 × 10^−6^
*x*_3_	1.3926	0.2405	5.7900	0.0022
*x*_1_*x*_2_	4.2282	0.2809	15.0540	2.3431 × 10^−5^
*x*_1_*x*_3_	−0.8748	0.2809	−3.1140	0.0264
*x*_2_*x*_3_	−0.3611	0.2809	−1.2860	0.2549
*x*^2^_1_	5.3117	0.4940	10.7540	0.0001
*x*^2^_2_	0.6467	0.4222	1.5320	0.1862
*x*^2^_3_	−2.3881	0.4222	−5.6560	0.0024
*B. thuringiensis* Al Hakam				
Intercept	−0.2778	0.5317	−0.5220	0.6127
Block (b)	0.1487	0.6584	0.2260	0.8259
*x*_1_	4.544	0.3822	11.8900	3.1850 × 10^−7^
*x*_2_	4.0575	0.3473	11.6830	3.7552 × 10^−7^
*x*_1_*x*_2_	3.9837	0.3883	10.2600	1.2560 × 10^−6^
*x*^2^_1_	1.5441	0.7735	1.9960	0.0739
*x*^2^_2_	2.6875	0.7264	3.7000	0.0041

Df = 1 for estimates.

#### *B. thuringiensis* Al Hakam on nylon

Test runs one and fourteen were difficult to fit. These runs were omitted and the model was refit. Interestingly, the number of days of treatment was not a significant predictor for this coupon (p = 0.664), so it was excluded from the model. A second order model with pure quadratic terms and two-way interactions was used. The F-test for lack of fit had a P-value of 0.698 which did not indicate lack of fit. R^2^ and adjusted R^2^ for the model were 0.9778 and 0.9644, respectively. Table 5 shows the parameter estimates, standard errors, t-values, and P-values. The fitted equation for inactivation of *B. thuringiensis* Al Hakam on nylon is *Ŷ* = −0.2778 + 0.1487*b* + 4.5440*x*_1_ + 4.0575*x*_2_ + 3.9837*x*_1_*x*_2_ + 1.5441*x*^2^_1_ + 2.6875*x*^2^_2_, where *Ŷ* is the log odds of spore inactivation. Figure 4 shows the response surface with block (b) fixed at its mean value of 0.2941.

### Wiring insulation

#### *B. anthracis* ∆Sterne on wiring insulation

Most observations for wiring insulation had complete inactivation of spores and could not be modeled using RSM. All runs from Table 1 had complete inactivation except for runs two, three, nine, and twelve. The notable thing about these four points is that they all had two or more predictors at the low setting; i.e., at least two of *x*_1_, *x*_2_, or *x*_3_ were equal to −1 for these four observations. This might be considered as the “back corner” of the predictor space. Excepting these four points, the model would be *Ŷ* = ∞ or, equivalently, prob(inactivation) = 1.

#### *B. thuringiensis* Al Hakam on wiring insulation

The results for *B. anthracis* ∆Sterne apply to *B. thuringiensis* Al Hakam.

### Polypropylene

#### *B. anthracis* ∆Sterne on polypropylene

**Figure 5:**
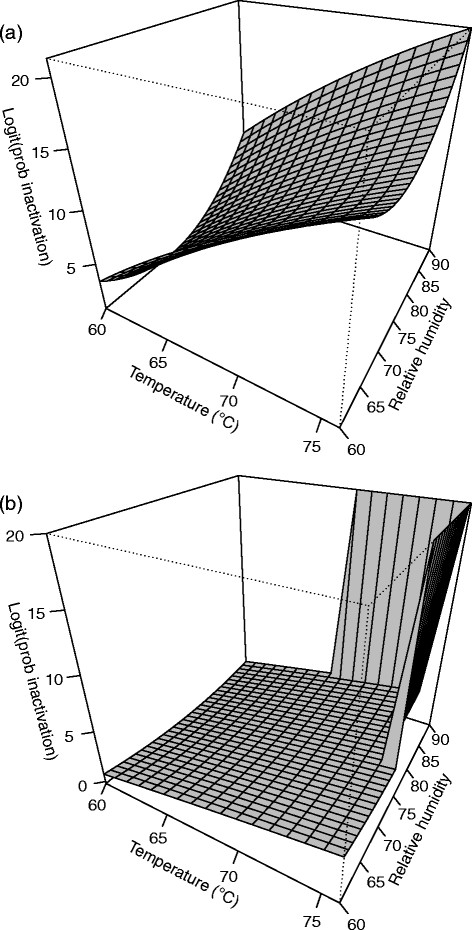
**Response surface plot for (a)** ***B. anthracis*** **∆Sterne and (b)** ***B. thuringiensis*** **Al Hakam on polypropylene at 4 days.**

Run number one, a center run, was an outlier and deleted from the analysis. A second order model with pure quadratic terms was used in this case. Table 6 shows that the significant pure quadratic term is relative humidity. Adding two-way interaction terms did not contribute significantly to the model (p = 0.641) and were not included. The F-test for lack of fit had a P-value of 0.187 which did not indicate lack of fit. R^2^ and adjusted R^2^ for the model were 0.8713 and 0.7811, respectively. Table 6 shows the parameter estimates, standard errors, t-values, and P-values. The fitted equation for inactivation of *B. anthracis* ∆Sterne on polypropylene is *Ŷ* = 6.4025 + 6.4256*b* + 6.9452*x*_1_ + 2.2431*x*_2_ + 4.3604*x*_3_ − 1.0017*x*^2^_1_ + 4.8915*x*^2^_2_ − 2.3429*x*^2^_3_, where *Ŷ* is the log odds of spore inactivation. Figure 5 shows a slice of the response surface at four days and block (b) fixed at its mean value of 0.3333.

**Table 6:** **Parameter estimates for** ***B. anthracis*** **∆Sterne and** ***B. thuringiensis*** **Al Hakam on polypropylene**

**Model term**	**Parameter estimate**	**Standard error**	**t-value**	**P-value**
*B. anthracis* ∆Sterne				
Intercept	6.4025	1.8516	3.4578	0.0061
Block (b)	6.4256	2.0000	3.2128	0.0093
*x*_1_	6.9452	1.1711	5.9306	0.0001
*x*_2_	2.2431	1.1711	1.9154	0.0844
*x*_3_	4.3604	1.1711	3.7234	0.0040
*x*^2^_1_	−1.0017	2.2678	−0.4417	0.6681
*x*^2^_2_	4.8915	2.2678	2.1570	0.0564
*x*^2^_3_	−2.3429	2.2678	−1.0331	0.3259
*B. thuringiensis* Al Hakam				
Intercept	1.4271	0.1529	9.3320	0.0002
Block (b)	0.7180	0.2202	3.2600	0.0224
*x*_1_	1.7227	0.1529	11.2640	9.6332 × 10^−5^
*x*_2_	−0.1465	0.1529	−0.9580	0.3822
*x*_3_	0.3641	0.1249	2.9160	0.0332
*x*^2^_1_	−0.0792	0.2823	−0.2810	0.7903
*x*^2^_2_	0.8467	0.2823	2.9990	0.0301
*x*^2^_3_	−0.5264	0.2777	−1.8960	0.1165

Df = 1 for estimates.

#### *B. thuringiensis* Al Hakam on polypropylene

Complete inactivation of *B. thuringiensis* Al Hakam spores was measured for test runs five, seven, fifteen, seventeen and nineteen. These data prevented the fitting of a second order model. Going beyond the usual RSM framework, however, a piecewise model was fitted using a second order model with pure quadratic terms for the points of incomplete activation and mapping the observations in the design space enclosed by runs five, seven, fifteen, and seventeen to ∞ or, equivalently, prob(inactivation) = 1. The region of incomplete inactivation was fitted as *Ŷ* = 1.4271 + 0.7180*b* + 1.7227*x*_1_ − 0.1465*x*_2_ + 0.3641*x*_3_ − 0.0792*x*^2^_1_ + 0.8467*x*^2^_2_ − 0.5264*x*^2^_3_, where *Ŷ* is the log odds of spore inactivation. Pure quadratic terms were significant for that part of the model. Table 6 shows that relative humidity is the significant second order term. Two-way interactions were not significant (p = 0.449). The F-test for lack of fit had a P-value of 0.452 which did not indicate lack of fit. Table 6 shows the parameter estimates, standard errors, t-values, and P-values. Figure 5 shows a slice of the response surface at four days and block (b) fixed at its mean value of 0.2308. We used *Ŷ* =20 instead of *Ŷ* = ∞ to keep the scale in the figure reasonable but still show the piecewise behavior of the model

(2) $$\hat{Y}=\left\{\begin{array}{l} \infty ,\mathrm{if}\left(x_{1}\geq 0,x_{2}\geq 1,x_{3}\leq x_{1},x_{3}\geq -x_{1}\right)\mathrm{or}\left(x_{1}\geq 1,x_{2}\geq 0,x_{3}\leq x_{2},x_{3}\geq -x_{2}\right)\\ 1.4271+0.7180b+1.7227x_{1}-0.1465x_{2}+0.3641x_{3}-0.0792x_{1}^{2}+0.8467x_{2}^{2}-0.5264x_{3}^{2} \end{array}\right)$$

A response with such a large jump is not the most desirable model and demonstrates the need for further experimentation using less extreme values of the predictors on polypropylene. Nevertheless, it contrasts the difference in behavior of polypropylene versus the other materials tested.

### Wet solution

#### *B. anthracis* ∆Sterne in wet solution

Most observations for wet solution had complete inactivation of spores, and could not be modeled using RSM. All *B. anthracis* ∆Sterne runs from Table 1 had complete inactivation except for runs three, six, and nine. The notable thing about these three points is that they all had temperature at the low setting.

#### *B. thuringiensis* Al Hakam in wet solution

All *B. thuringiensis* Al Hakam test runs from Table 1 had complete inactivation except for runs one, three, six, nine, and twelve. Except for run one, a center point, these observations had temperature at the low setting.

### Contour plots in two dimensions

**Figure 6:**
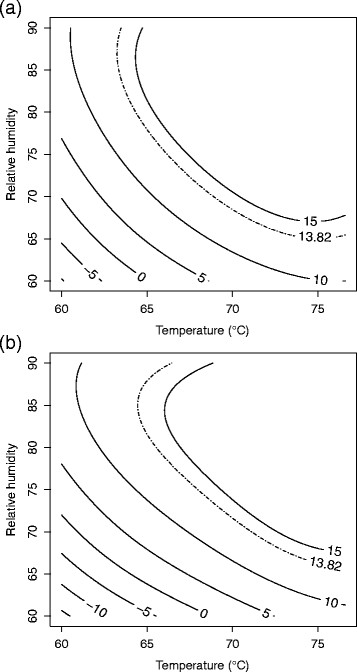
**90% lower confidence contour for spore inactivation of (a)** ***B. anthracis*** **∆Sterne and (b)** ***B. thuringiensis*** **Al Hakam on APC.**

**Figure 7:**
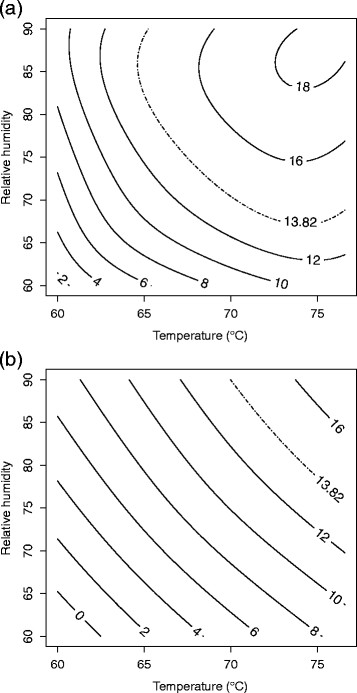
**90% lower confidence contour for spore inactivation of (a)** ***B. anthracis*** **∆Sterne and (b)** ***B. thuringiensis*** **Al Hakam on antiskid.**

**Figure 8:**
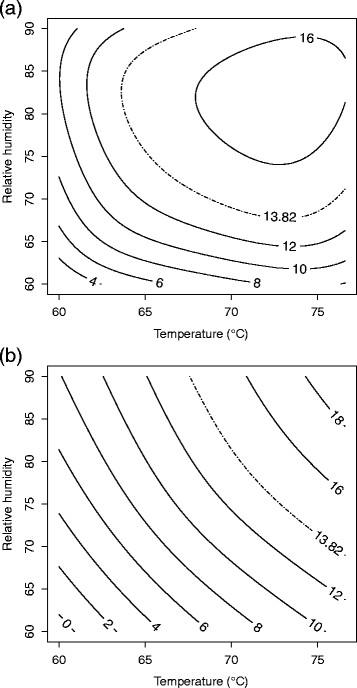
**90% lower confidence contour for spore inactivation of (a)** ***B. anthracis*** **∆Sterne and (b)** ***B. thuringiensis*** **Al Hakam on InsulFab.**

**Figure 9:**
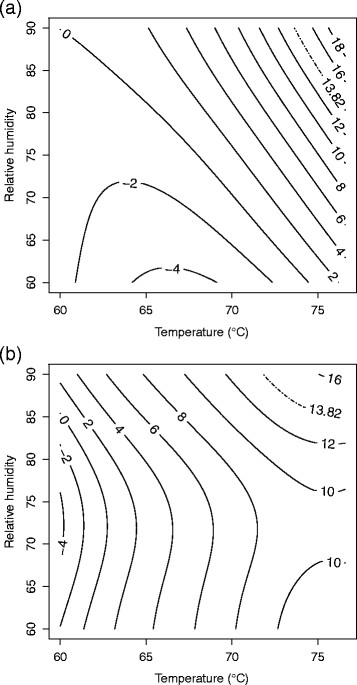
**90% lower confidence contour for spore inactivation of** ***B. anthracis*** **∆Sterne on (a) nylon and (b) polypropylene.**

Decontamination requirements were a ≥6-log (10^6^) spore inactivation out of a ≥6-log (10^6^) spore challenge (Buhr et al. [2012b]). This translates into a probability of spore inactivation equal to 0.999999 or a log odds of spore inactivation equal to $\log\left(\frac{0.999999}{1-0.999999}\right)\approx 13.82$. The lower bound from a two-sided 90% confidence surface for the log odds of inactivation was used for the contour plots (Myers and Montgomery [2002]). In cases where a model could be fit using Equation 1, a contour plot for temperature vs. relative humidity is given in original units with days fixed at 4. (The plot for *B. thuringiensis* Al Hakam on nylon was omitted since the region of ≥6-log spore inactivation was barely visible on the graph.) These are given in Figures 6, 7, 8, and 9.

## Discussion

Control-driven test method improvements and the use of multiple independent spore preparations with a single protocol useful for both *B. anthracis* ∆Sterne and *B. thuringiensis* Al Hakam (Buhr et al. [2012b]) allowed for the application of a statistically based experimental design, specifically RSM. This use of RSM permitted subsequent mathematical analysis and modeling of the response, generating a predictive capability valuable to potential users of hot, humid air decontamination technology. These advancements in data analyses and interpretation, when coupled with decreased test time and cost (Buhr et al. [2012b]), serve to better support the transition of decontamination technologies from the laboratory to the field.

In all modeled strain-material pairs but one, all three predictors (temperature, relative humidity, and time) were significant and each had an effect on spore inactivation. The exception was *B. thuringiensis* Al Hakam spores dried onto nylon webbing material, where time was not significant. Interestingly, for *B. anthracis* ∆Sterne on two materials (InsulFab and polypropylene), relative humidity was the most significant second order pure quadratic term.

The most difficult materials to model were nylon webbing and polypropylene (dried plastic tubes) where a piecewise modeling strategy was required to fit *B. thuringiensis* Al Hakam on polypropylene. Nylon was the only material for which multiple runs were omitted based on examination of residual plots. Hot, humid air was least effective at inactivating spores on nylon. This may be due to the porous and/or hydrophobic properties of nylon.

For wet spores and those dried onto wiring insulation, the majority of test runs showed complete inactivation. For wet spores of both strains, the cases without complete inactivation were at the lowest setting for temperature with only one exception. For spores of both strains on wiring insulation, the conditions without complete inactivation all had two or more predictors at the lowest settings.

The mathematical models were cross-checked with published spore survival results for high purity spores of *B. anthracis* ∆Sterne and *B. thuringiensis* Al Hakam (Buhr et al. [2012b]). The published data set consisted of total of 210 combinations of strain/substrate/temperature/relative humidity/time including the six substrates and the wet control (seven total). Data was compiled from 1,330 test samples and 1,330 room temperature controls over 19 independent test runs (Buhr et al. [2012b]). These published data were cross-checked with the contour graphs. Spore inactivation data of less than 6-log was below the 90% lower statistical confidence line (higher practical confidence), and spore inactivation data of greater than 6-log was above the 90% lower statistical confidence line. Thus each spore inactivation/decontamination datum was located at a logical position in the contour graphs. Both the published decontamination results and the mathematical models showed that *B. anthracis* ΔSterne and *B. thuringiensis* Al Hakam spores were similarly decontaminated, although *B. thuringiensis* Al Hakam spores survived slightly better on some substrates, such as polypropylene, under the mildest temperature/relative humidity/time conditions.

This work demonstrates the use of statistical methods to design biological decontamination experiments at the limits of decontamination technology and to analyze the resultant data. The fitted equations specific to strain and material permit a broad range of end-users with varying requirements and constraints the ability to predict the success of hot, humid air treatments on specific materials based on achievable temperature and relative humidity conditions and permissible decontamination times.

## Competing interests

A patent application has been submitted on behalf of the United States Navy regarding the mathematical models in order to protect the United States government’s investment in this technology.
